# Iatrogenic Air Embolisms During Endovascular Interventions: Impact of Origin and Number of Air Bubbles on Cerebral Infarctions

**DOI:** 10.1007/s00062-023-01347-2

**Published:** 2023-09-04

**Authors:** Tabea C. Schaefer, Svenja Greive, Stine Mencl, Sabine Heiland, Martin Kramer, Markus A. Möhlenbruch, Christoph Kleinschnitz, Martin Bendszus, Dominik F. Vollherbst

**Affiliations:** 1 Department of Neuroradiology, University Hospital Heidelberg, Heidelberg, Germany; 2 Clinic for small animals, Justus-Liebig-University Gießen, Gießen, Germany; 3 Department of Neurology and Center for Translational Neuro- and Behavioral Sciences (C-TNBS), University Medicine Essen, Essen, Germany

**Keywords:** Gas embolism, TEVAR, TAVI, Cerebral infarction, Endovascular interventions

## Abstract

**Purpose:**

Cerebral infarctions caused by air embolisms (AE) are a feared risk in endovascular procedures; however, the relevance and pathophysiology of these AEs is still largely unclear. The objective of this study was to investigate the impact of the origin (aorta, carotid artery or right atrium) and number of air bubbles on cerebral infarctions in an experimental in vivo model.

**Methods:**

In 20 rats 1200 or 2000 highly calibrated micro air bubbles (MAB) with a size of 85 µm were injected at the aortic valve (group Ao), into the common carotid artery (group CA) or into the right atrium (group RA) using a microcatheter via a transfemoral access, resembling endovascular interventions in humans. Magnetic resonance imaging (MRI) using a 9.4T system was performed 1 h after MAB injection followed by finalization.

**Results:**

The number (5.5 vs. 5.5 median) and embolic patterns of infarctions did not significantly differ between groups Ao and CA. The number of infarctions were significantly higher comparing 2000 and 1200 injected MABs (6 vs. 4.5; *p* < 0.001). The infarctions were significantly larger for group CA (median infarction volume: 0.41 mm^3^ vs. 0.19 mm^3^; *p* < 0.001). In group RA and in the control group no infarctions were detected. Histopathological analyses showed early signs of ischemic stroke.

**Conclusion:**

Iatrogenic AEs originating at the ascending aorta cause a similar number and pattern of cerebral infarctions compared to those with origin at the carotid artery. These findings underline the relevance and potential risk of AE occurring during endovascular interventions at the aortic valve and ascending aorta.

**Supplementary Information:**

The online version of this article (10.1007/s00062-023-01347-2) contains supplementary material, which is available to authorized users.

## Introduction

Iatrogenic air embolisms (AEs) are a well-known and feared potential cause of cerebral infarctions during endovascular interventions at the brain-supplying vessels or the cerebral vessels themselves, such as carotid artery stenting, mechanical thrombectomies, and embolization treatments of aneurysms or vascular malformations [1]. In endovascular cardiothoracic interventions, such as thoracic endovascular aortic repair (TEVAR) and transcatheter aortic valve implantation (TAVI), the risk of AE-related cerebral infarctions is known [2, 3] but the relevance is a matter of debate and compared to interventions at the cervical or cerebral vessels, it is often less considered and less scientifically investigated.

The presence of AEs in the brain can have various effects, reaching from small punctate infarctions without any clinical sequalae or only subtle neurocognitive deficits to potentially fatal ischemic strokes [4–7]; however, also small infarctions in eloquent regions can cause disabling symptoms. The incidence of clinically silent brain infarctions after cardiac surgery is up to 46% and reaches up to 67% after the treatment of intracranial aneurysms with flow diverting stents [8, 9]. These infarctions might also be caused by other microemboli, such as small thrombi; however, AE are regarded to be the main contributor to these ischemic lesions [1, 10–12], which is also supported by observations that the vast majority of embolic phenomena during carotid angiograms occur during catheter flushing and injection of contrast material [13]. Most AEs which are clinically relevant occur in the arterial system; however, there are also venous AE which can pass or bypass the lungs, which normally filters the air and cause embolisms on the arterial side [14].

The effects of these AEs are supposed to depend on various factors, such as the size of the air bubbles, the injection speed, and most importantly, the volume of induced air and the origin of the AE [4, 6, 7]. Until now, no study systematically assessed the influence of the point of origin and of the amount of air bubbles on the frequency and distribution of cerebral infarctions.

Studies which assessed the number of air bubbles leading to cerebral AE during various cardiosurgical interventions found that these bubbles are so-called micro air bubbles (MABs) with a diameter smaller than 100 µm [15, 16]. Therefore, this study was focused on these MABs.

The high and still increasing number of catheter-based interventions in the neurological and cardiothoracic fields and the high frequency of AEs underlines the importance of the understanding of the pathophysiology of AE occurring in these procedures.

The aim of this study was to investigate the influence of application site and air bubble number on cerebral infarctions in an experimental in vivo model.

## Material and Methods

### Generation and Detection of Air Bubbles

The MABs which were injected were generated and automatically assessed as described previously [17]. In brief, using a microfluidic controller and a specifically designed microfluidic channel system, MABs were generated in 80% contrast agent diluted in normal saline. We aimed to produce MAB with a target size of 85 µm, as this is a common size for iatrogenic AE in endovascular interventions in clinical practice [15, 16, 18].

### Animal Procedure

All experiments were performed in accordance with the Guide for the Care and Use of Laboratory Animals (Directive 2010/63/EU of the European Parliament). State Animal Care and ethics committee approval was obtained (registry number: 35-9185.81/G-183/20).

In this study 27 male and female Wistar rats (Janvier Laboratories, Le Genet-St-Isle, France) with an average body weight of 375 g were used. The animals were randomly allocated to the study groups, while the distribution of the sexes in each study and control group was balanced.

The rat was chosen as animal model because of its practicability as an experimental animal and as the cerebral blood supply in rats is similar to humans [19]. In both species, a vascular network, located at the base of the brain, is formed from the internal carotid arteries and the basilar artery, forming an arterial circulus.

The animals were anesthetized with an intraperitoneal injection of 100 mg/kg ketamine (Ketamin 10%, Pharmanovo, Dobl, Austria) and 5 mg/kg xylazine (Xylariem®, Ecuphar GmbH, Greifswald, Germany). During the MRI the rats were anesthetized in a special anesthetic induction chamber with an initial dose of 4% isoflurane. A 26 G tail vein catheter was inserted before the MRI. During the measurement, inhalation anesthesia was obtained with an anesthetic mask to maintain a dose of 2% isoflurane. The animals were positioned on a heating pad to maintain a constant body temperature. They were breathing spontaneously and were monitored during the MRI with a breathing surface pad checked by an in-house developed LabView program (National Instruments, Munich, Germany).

At the end of the experiments, after 30 min of circulation, the animals were finalized by an intraperitoneal injection of a ketamine/xylazine overdose (200 mg/kg ketamine and 10 mg/kg xylazine). During the final heart beats, a perfusion with 50 ml of Dulbecco’s phosphate-buffered saline (gibco by life technologies, Bleiswijk, Netherlands) and afterwards 50 ml of 4% paraformaldehyde (PFA; Carl Roth GmbH + Co. KG, Karlsruhe, Germany) was performed to enable histopathological examination. The brain was then removed and fixed in 4% PFA for 3 days. Afterwards the sample was kept in 30% Saccharose (Carl Roth GmbH) for 48 h, were embedded in Tissue-Tek® O.C.T.™ (Sakura Finetek Europe B.V., Umkirch, Germany), and stored at −80 °C. For histology or fluorescent staining brains were cut into 10 µm thick coronal sections with a cryostat (Leica Biosystems, Nußloch, Germany) and stored at −20 °C.

### Endovascular Procedure

For vascular access the femoral artery or vein was surgically exposed, followed by an incision of the target vessel. A 26 G peripheral catheter (BD Neoflon, Heidelberg, Germany) was then introduced into the femoral artery (groups Ao and CA) or vein (group RA), and a 0.014-inch microguidewire (Traxcess 14; MicroVention, Aliso Viejo, USA) was inserted and positioned within the abdominal aorta or the inferior vena cava under fluoroscopic guidance (ARTIS icono or SIREMOBIL Compact L; Siemens Healthineers, Erlangen, Germany). After removing the 26 G catheter, a 156 cm/0.0165-inch microcatheter (Headway Duo; MicroVention) was inserted over the guidewire. The guidewire was then removed and the microcatheter was manually flushed with saline to clear the catheter. A continuous flush was then connected to the microcatheter using a pressure flushing system. The microcatheter was then positioned within the aorta at the level of the aortic valve (group Ao), the left common carotid artery (CCA; group CA) or the right atrium (group RA) under fluoroscopic guidance using the 0.014-inch guidewire. For injection of the MABs, the hub of the microcatheter was connected to the outlet tube of the microfluidic channel system using a manually modified syringe-catheter interface adapter. Afterwards either 1200 MABs (groups Ao-1200 and CA-1200) or 2000 MABs (groups Ao-2000, CA-2000, and RA-2000) were injected into the vascular system (duration of injection: 5 s; injection speed: 0.1 ml/s). In the control groups, the microcatheter was flushed with saline. After the procedure, the microcatheter was removed, the vessel was ligated, and the skin was stitched up with a continuous suture. In one additional animal, which was not part of any study group, we performed diagnostic angiographies, including rotational angiographies with subsequent three-dimensional reconstructions, with the microcatheter being positioned in the ascending aorta or the common carotid artery, as injection of contrast agent was avoided in the study animals to avoid producing more than the intended iatrogenic AEs. This experiment served for the illustration of the sites of MAB application and for the understanding of the hemodynamic flow of the injected liquids and MABs.

### MRI

Magnetic resonance imaging (MRI) was performed using a 9.4 T small animal MR system (Bruker Biospec 94/20; Bruker Biospin, Ettlingen, Germany) 1h after the endovascular procedure using the following sequences: time-of-flight angiography, T2-weighted sequences, diffusion-weighted imaging (DWI) sequences, T1-weighted sequences before and after the application of contrast agent, and susceptibility-weighted imaging. T2-weighted sequences were performed as the first and last sequence to monitor any change in size or demarcation of infarctions and to detect infarctions which might develop during the acquisition. The detailed MRI protocol is presented in the Supplemental Material.

### Histological Work-up

For all imaging of histology and fluorescence brain sections a Leica DM8i (Leica Microsystems, Germany) inverse microscope was used.

#### Hematoxylin and Eosin Stain

Cryosections were dried for 15 min at room temperature, before the embedding medium was removed with distilled water for 2 min. Hematoxylin and eosin solutions (#T865.2 and #X883.2 Roth, Germany) were used for the staining. In short, sections were incubated for 10 min in hematoxylin solution, washed for 15 min with warm and running tap water, followed by 2 min wash with aqua dest, and 1 min staining in eosin solution. Finally, we washed with running tap water, incubated the sections shortly in 70, 95 and 100% ethanol, twice for 2 min in Roticlear (#VA538.1; Roth, Germany), and mounted all sections with Cytoseal (#8312‑4, ThermoFisher, Germany).

#### Cresyl Violet Stain

Cryosections were dried for 15 min at room temperature, before the embedding medium was removed with distilled water for 2 min, followed by 5 min staining in 0.5% Cresyl Violet (in acetic acid and distilled water) at 60 °C. Then we washed twice with aqua dest, differentiated shortly in 95% ethanol, incubated for 5 min in 100% ethanol, incubated twice for 10 min in Roticlear (#A538.5 Roth, Germany) and mounted all sections with Cytoseal (#8312‑4, ThermoFisher, Germany).

#### Immunofluorescence

Cryosections were dried for 10 min at room temperature, fixed for 10 min in 10% formalin (#HT501128-4L, Sigma Aldrich, Germany). Embedding medium was removed with 0.02% Tween20 (T; #P1379-100ML, Sigma Aldrich, Germany) in tris buffered saline (TBS) twice for 5 min. All unspecific binding sites were blocked for 1 h at room temperature in 10% normal donkey serum (#S30-100ML, EMD Milipore, Germany), 1% bovine serum albumin (#0163.2, Roth, Germany) in TBST, followed by overnight incubation of the 1st antibody diluted as indicated in 1% bovine serum albumin in TBST at 4 °C. After 30 min at room temperature 1st antibody was carefully washed away with 4 repetitions of 5 min TBST. The 2nd antibody (A21247; A31571; A21434; A31572; A21432; Invitrogen and 703-545-155; Jackson ImmunoResearch) was diluted 1:500 in 1% bovine serum albumin in TBS, DAPI (300 nM, #D9542-5MG, Sigma Aldrich, Germany) was added, and after 1 h incubation at room temperature and 4 times 5 min wash in TBS, all sections were mounted with Dabco (#0718.1, Roth, Germany) in Mowiol (#0713.1 Roth, Germany). TUNEL staining (In Situ Cell Death Detection Kit, TMR red; #12156792910 Roche; Merck Germany) was applied according to the manual after 2nd antibody and before mounting with Dabco/Mowiol.

## Study Groups

**Fig. 1:**
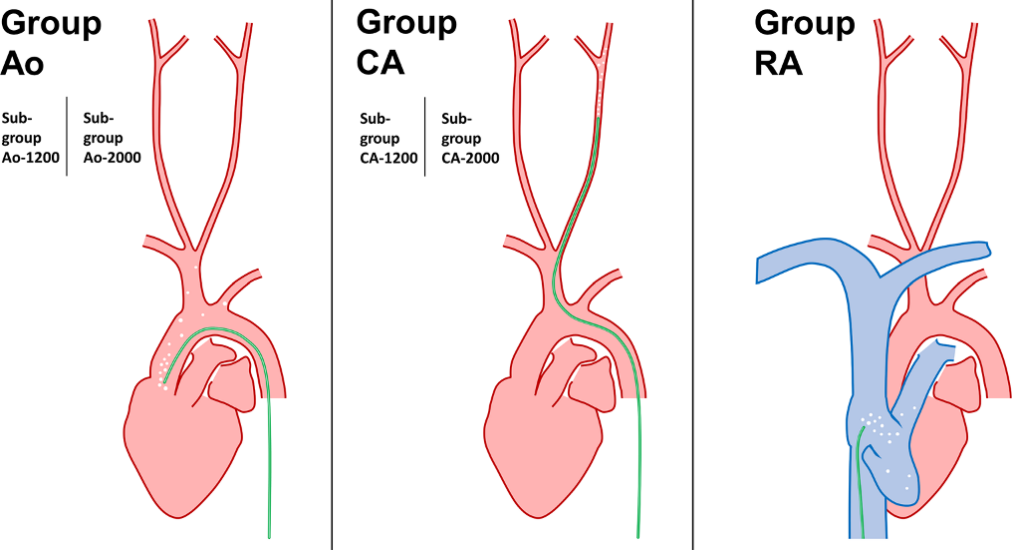
Illustration of the study groups. Using a microcatheter (illustrated in *green*), micro air bubbles (*white dots*) were injected into the origin of the aorta (group Ao, left diagram), the left common carotid artery (group CA, middle diagram) or into the right atrium (group RA, right diagram). For groups Ao and CA, either 1200 or 2000 bubbles were injected (subgroups Ao-1200 and CA-1200, Ao-2000 and CA-2000), while in group RA there was no subgroup (2000 bubbles were injected into the right atrium)

A total of 27 rats were used in this study with equal sex distribution in each study- and control group. Of these animals, 26 were allocated to the study groups (4 animals per study group) and control groups (3 animals per control group), and 1 additional animal was used for comprehensive diagnostic angiographies as described above. The study groups are illustrated in Fig. 1.

The following study groups were defined according to the origin of the MABs and the number of injected MABs: Ao-1200 (injection of 1200 MABs into the ascending aorta), Ao-2000 (injection of 2000 MABs into the ascending aorta), CA-1200 (injection of 1200 MABs into the left CCA), CA-2000 (injection of 2000 MABs into the left CAA), RA-2000 (injection of 2000 MABs into the right atrium) with 4 animals per groups, respectively. In 6 animals, the microcatheter was positioned within the aorta (*n* = 3) or the CCA (*n* = 3) with subsequent injection of the same volume of diluted contrast agent without MABs, serving as control groups.

### Study Goals

The number of infarctions, the volume of the respective infarctions, the total infarction volume per animal (the sum of all infarction volumes in one animal), and the location of these infarctions were assessed in MRI using T2-weighted and DWI sequences. Measurement of the volume of the infarctions was performed via semi-automated segmentation of each infarction in T2-weighted 3D sequences using the Medical Imaging Interaction Toolkit (MITK; German Cancer Research Center, DKFZ, Heidelberg, Germany). The MRI sequences were analyzed regarding pathological changes, such as visualization of MABs or vessel occlusions. Histopathological examinations aimed to assess early findings of ischemia and immune response.

### Statistics

GraphPad Prism (La Jolla, USA; version: 7.04) was used for this statistical analysis. Quantitative data are presented as medians with interquartile range (median (1st quartile; 3rd quartile)), except for the descriptive statistics of the produced and detected MABs. To evaluate statistical differences between the study groups (group Ao vs. group CA; groups Ao1200+CA1200 vs. groups Ao2000+CA2000), the Mann-Whitney test was performed. A *p*-value of 0.05 was defined as the threshold for statistical significance.

## Results

### Size of the Produced MAB

A total number of 25,600 highly calibrated MABs was created. The average diameter of all generated MABs was 85.2 µm (mean)/85.3 µm (median) with a standard deviation of 2.1 µm and a coefficient of variation of 0.02.

### Imaging Findings

**Fig. 2:**
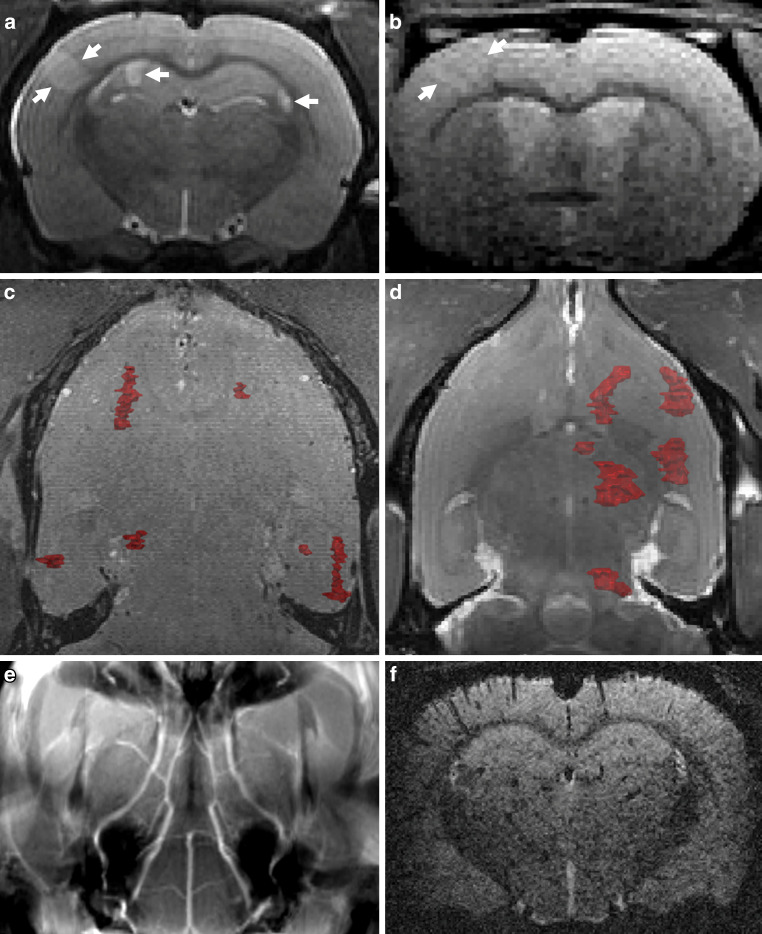
Magnetic resonance imaging findings. In an axial slice of T2-weighted sequences (**a**), shown in an example animal of group Ao-1200, infarctions were visible as well-demarcated hyperintense lesions (*white arrows*). In diffusion-weighted imaging (**b** axial view) these lesions correlated to areas of restricted diffusion. Colored volume renderings of the infarctions for example animals of group Ao-2000 (**c**, coronal view) and of group CA-2000 (**d** coronal view) demonstrate bilateral smaller infarctions in both hemispheres for group Ao and unilateral larger infarctions for group CA. No vascular occlusions were visible in the time-of-flight angiography (**e** coronal maximal intensity projection) and no circumscribed areas of signal loss, consistent with air bubbles, in susceptibility-weighted sequences (**f** axial view)

**Fig. 3:**
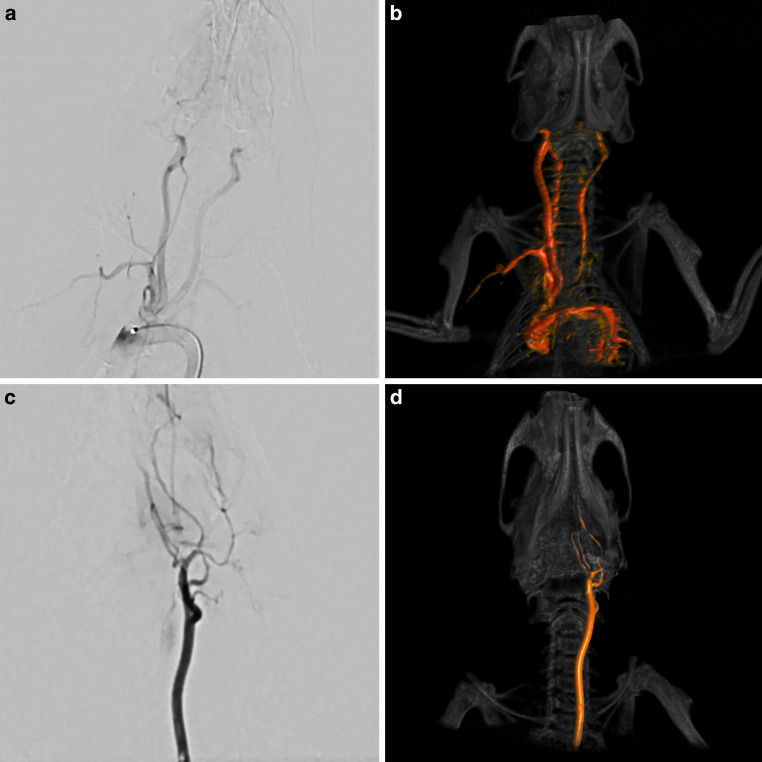
Angiography of the sites of air bubble application. Conventional digital-subtraction angiographies (**a**, **c**) and volume-renderings of rotational angiographies (**b**, **d**) were performed with the microcatheter positioned either in the proximal aortic arch (**a**, **b**) or the left common carotid artery (**c**, **d**) in an additional animal. Injection of contrast medium into the proximal aorta led to opacification of the supra-aortic vessels, and to a lesser extent of the descending aorta, which illustrates the relatively high number of cerebral infarctions caused by the AE out of this position. Injection into the left common carotid artery only opacified ipsilateral craniocervical vessels, consistent with the exclusively unilateral infarctions

Representative MRI findings are presented in Fig. 2 and illustrative angiography findings are presented in Fig. 3.

In MRI, several well-demarcated T2-hyperintense regions with correlating diffusion restriction were observed, consistent with acute ischemic infarctions. No macroscopically visible occlusion of cerebral vessels was observed in TOF-angiography and no macroscopically recognizable air could be detected in susceptibility-weighted sequences.

Diagnostic angiographies with the catheter positioned in the proximal aorta led to opacification of the supra-aortic vessels, predominantly on the right side and, to a lesser extent of the descending aorta. Injection of contrast agent into the left common carotid artery only opacified ipsilateral craniocervical vessels.

### Number and Volume of Cerebral Infarctions

**Fig. 4:**
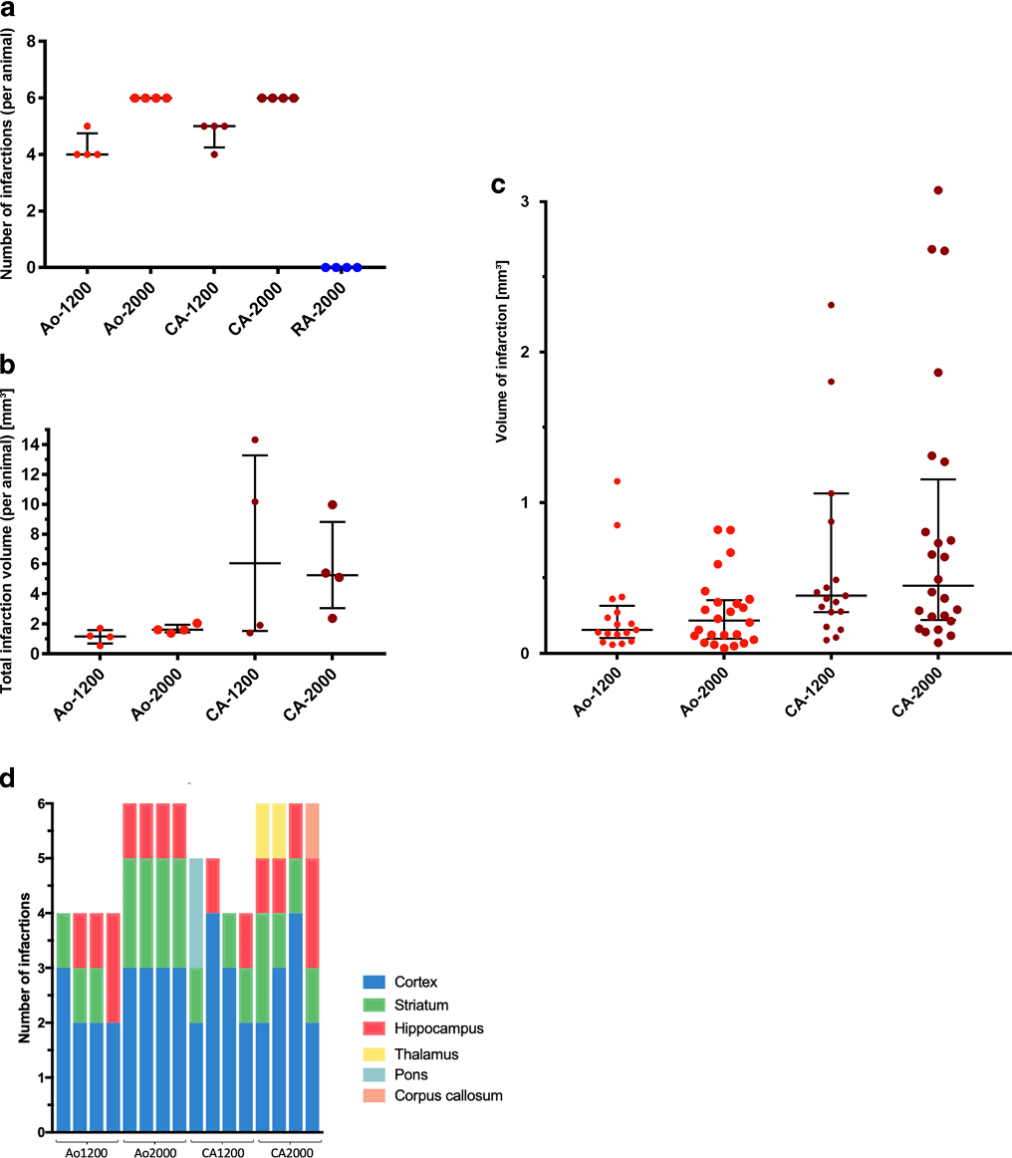
Summary of the number, volume, and distribution of infarctions. Number of infarctions (**a**), total infarction volume (**b**) and volume of the single infarctions (**c**) are illustrated as scattered dot plots (*lines* indicating median and interquartile range). In (**c**), two data points of sub-group CA-1200 (8.96 and 8.99 mm^3^) are outside the axis limit. The distribution of the infarctions is shown as bar graph (**d**)

All animals of groups Ao and CA developed between 4 and 6 infarctions, detectable in MRI. There was no significant difference between the number of infarctions comparing group Ao and group CA (medians 5.5 vs. 5.5; *p* = 0.77). The number of infarctions was significantly higher comparing 2000 and 1200 injected MABs (6 vs. 4.5; *p* < 0.001). In group RA as well as in the control groups, no infarction was detected in MRI. The volume of the single infarctions was significantly higher in group CA compared to group Ao (0.41 mm^3^ vs. 0.19 mm^3^; *p* < 0.001). Also, the total infarction volume was higher in group CA (5.25 mm^3^ vs. 1.50 mm^3^; *p* = 0.003). Comparing 1200 vs. 2000 injected MABs, the volume of infarction and the total infarction volume did not differ significantly and 2 relatively large infarctions with 8.96 and 8.99 mm^3^ volume were detected in 2 animals in group CA-1200. The summary of the quantitative results is presented in Table 1 and Fig. 4.

**Table 1:** Summary of the study groups and of the quantitative results

*Study group*^a^	*Ao*		*CA*		*RA*	*Control*		*p‑value*^d^* (Ao vs. CA)*
Number of infarctions	5.5 (4; 6)		5.5 (5; 6)		0 (0; 0)	0 (0; 0)		0.77
Volume of infarction^b^ [mm^3^]	0.19 (0.10; 0.35)		0.41 (0.25; 1.06)		–	–		< 0.001
Total infarction volume^c^ [mm^3^]	1.50 (1.16; 1.69)		5.25 (2.03; 10.12)		–	–		0.003
*Subgroup*^a^	*Ao-1200*	*Ao-2000*	*CA-1200*	*CA-2000*	*(2000)*^e^	*Ao‑0*	*CA‑0*	*p‑value*^f^* (1200 vs. 2000)*
Number of infarctions	4 (4.0; 4.75)	6 (6.0; 6.0)	5 (4.25; 5.0)	6 (6.0; 6.0)	0 (0; 0)	0 (0; 0)	0 (0; 0)	< 0.001
Volume of infarction^b^ [mm^3^]	0.16 (0.10; 0.32)	0.22 (0.10; 0.36)	0.38 (0.28; 1.06)	0.45 (0.22; 1.16)	–	–	–	0.95
Total infarction volume^c^ [mm^3^]	1.18 (0.69; 1.59)	1.61 (1.45; 1.95)	6.05 (1.54; 13.28)	5.25 (3.06; 8.83)	–	–	–	0.38

Quantitative data are presented as medians with interquartile range (median (1st quartile; 3rd quartile))

*Ao* aorta (level of aortic valve), *CA* carotid artery, *RA* right atrium

^a^ Letters indicate the site of air bubble application; numbers indicate the number of injected micro air bubbles

^b^ Volume of the single infarctions

^c^ Total infarction volume per animal

^d^ Group Ao vs. Group CA, Mann-Whitney test

^e^ There was no sub-group in Group RA since no infarctions were detected after injection of 2000 MABs

^f^ Groups Ao1200 + CA1200 vs. Groups Ao2000 + CA2000, Mann-Whitney test

### Infarction Pattern

All animals of group Ao showed infarctions in both hemispheres, whereas in group CA, only ipsilateral infarctions were observed. All animals developed cortical infarctions and 14 of 16 animals (87.5%) developed infarctions in the striatum and hippocampus, respectively. There was a similar and embolic pattern of infarctions across the study groups with most infarctions located in the cortex, the striatum, and the hippocampus. Infarctions in the thalamus, pons, and corpus callosum were only observed in group CA.

### Histopathological Findings

**Fig. 5:**
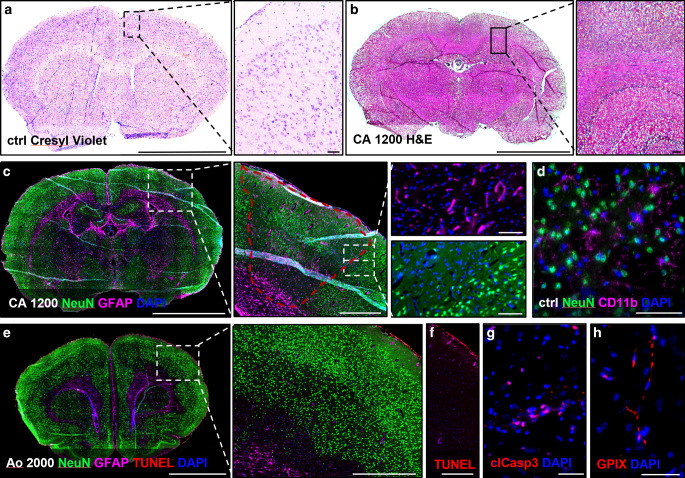
Representative histopathological findings after experimental AE. Rat brains were sectioned and stained for histochemistry analysis with Cresyl violet (Nissl stain) for neuronal pathology (**a**), or with hematoxylin and eosin stain for tissue pathology (**b**). Magnifications of the cortical area or the hippocampus (*black square* and *dotted lines*) are shown left of (**a**) and (**b**). After immunofluorescence staining (**c**–**h**) showing neurons (NeuN) and active astrocytes (GFAP) we detected areas with reduced neuronal cell number (*red dotted line* in the magnification), while other cell types were still present (divided high magnification). Macrophages/microglial cells (CD11b) were not different between the experimental groups (**d**). For the visualization of neuronal apoptotic cell death in infarcted areas, we did co-staining (**e**) of neurons (NeuN), active astrocytes (GFAP) and apoptotic cells (TUNEL). In a magnification of the TUNEL stain (**f**), without the neuronal (NeuN) or cell nuclei (DAPI) channel, the location of apoptotic cells was restricted to the most peripheral area. An early cell death protein (cleaved caspase 3) was found in some brain cells but did not show differences between the treatment groups (**h**). To visualize thrombotic vessels, platelets (GPIX) were stained, but no occluded vessel was detected. Scale bars: 5 mm (whole brains); 100 µm (magnifications); 50 µm (**g**, **f**, **h**)

Representative histochemical and immunofluorescence-stained rat brain tissues after AE are shown in Fig. 5.

With histochemical methods such as cresyl violet, a specific neuronal stain, or with hematoxylin and eosin, a non-specific tissue stain, we could not detect any infarcted areas or other tissue pathologies. Therefore, we switched to more specific immunofluorescence staining against neuronal cells (NeuN), activated astrocytes (GFAP), cell nuclei (DAPI), macrophages/microglia (CD11b, Iba1), neutrophil granulocytes (Ly6G), dying cells (TUNEL, cleaved caspase 3, c‑Jun, cytochrome C), and platelets (GPIX).

Several larger infarcts were visualized with the neuronal NeuN staining as the NeuN signal was absent in the infarcted area, but GFAP and nuclei were still detectable. We used two different microglial markers along with NeuN and GFAP to image the infarcted areas; however, no difference was seen in CD11b between the experimental groups, while Iba 1, a marker for activated microglia, was absent at this early time-point after AE. Another immune cell population with a fast reaction to ischemic brain injury ts neutrophil granulocytes, but we could not find them inside the brain tissues at this time-point after AE.

To detect apoptotic cell death, a TUNEL assay was performed together with NeuN and GFAP. TUNEL-positive cells were mainly found in the marginal cell layer of the brain, most likely showing cell death that occurred through the processing of the brain tissue. Other cell death markers (c-June and cytochrome c) were not detectable.

We stained platelets (GPIX) to detect occluded vessels but could rarely find a positive signal.

## Discussion

In this study, the impact of the point of origin of AE and the number of MABs on the number, size and distribution of cerebral infarctions was systematically analyzed in an experimental in vivo model. It could be demonstrated that the number and the pattern of hemispheric distribution of brain infarctions is similar between AEs originating at the proximal aorta and the CCA. This is the first study analyzing iatrogenic AE using a catheter-based experimental in a vivo small animal model resembling endovascular procedures in humans.

As indicated in the introduction, in interventions involving the cervical brain supplying vessels or the cerebral vessels themselves, AE are well-known and highly considered, as the vessels which are catheterized and treated are, in most cases, directly supplying the brain. For the prevention of AEs during these neurointerventions, catheters which are inserted into the cerebral vascular usually are constantly flushed, and syringes which are filled with contrast agent or saline for injection are carefully assessed, and, if necessary, cleared from macroscopically visible air bubbles. In cardiothoracic interventions, the site of action, the heart, the aortic valve, and the ascending aorta,is distant from the brain-supplying arteries, which might explain why AEs are comparably less considered as a potentially harmful risk in these interventions. Several recent studies, predominantly on TEVAR, studied the occurrence of AE during these procedures and showed that relevant volumes of air are generated during the deployment of the stent grafts [16, 20]. Furthermore, specific flushing techniques to reduce this air were investigated in recent years [21, 22].

One might expect that many MABs originating from the heart and the ascending aorta follow the aorta and are flushed into the peripheral vasculature, where AE are less relevant; however, as illustrated in the angiographies (see Fig. 3) and based on the distribution of the detected cerebral infarctions, we found that a substantial number of MABs which originate from the proximal aorta enter the cerebral circulation. Accordingly, we observed a similar number of infarctions after the injection of MABs into the proximal aorta or into the common carotid artery. Even though the volume of the infarctions and the total infarction volume was lower if the MABs were not injected directly into the cerebral vasculature, this finding underlines the relevance of AE in cardiothoracic endovascular procedures. Also, the hemispheric distribution of infarctions was similar with most infarctions located in cortical regions. Infarctions in deeper cerebral regions, such as the thalamus and pons were only detected after injection of MABs directly into the CCA. A potential explanation for this finding is that MABs which enter the cerebral circulation via the aorta are distributed according to the blood flow, and thus cause infarctions in cerebral regions which are supplied by the larger cerebral arteries. If MABs are injected into the CCA with a certain pressure, also smaller and non-major arteries can be reached, causing infarctions in deeper regions of the brain.

The impact of the number of iatrogenic MABs on the number, volume or distribution of cerebral infarctions was not systematically assessed before. We could demonstrate that the number of MABs has a major impact on the size of brain infarctions. The injection of 2000 vs. 1200 MABs caused larger cerebral infarctions with a correspondingly larger total infarction volume, while the number of infarctions was similar. The similar number of infarctions, despite a substantial difference in the number of MABs, might also be caused by the fusion of multiple MABs to a few larger bubbles as mentioned below.

Venous AE causing cerebral infarctions is a rare phenomenon, most frequently caused by a PFO or by intrapulmonary shunting, called paradoxical air embolism [4, 14]. In this small animal model, the selected number and size of air bubbles in the venous system did not cause cerebral infarctions. As a PFO is not known to occur in the rat [23], multiple AEs seem not to be able to bypass the lungs or exceed the pulmonary filter capacity of the lungs.

Also, in the control group, no cerebral infarctions were observed. We can thus conclude that the detected infarctions are in fact caused by the MABs and not by the catheterization of the aorta or the cerebral vessels.

A noteworthy finding of this study is that a high number of injected MABs (either 1200 or 2000) caused only a relatively low number of 4–6 infarctions in the brain. This phenomenon can be explained by the fusion of multiple smaller MABs to form larger air bubbles. Similar findings, but to a different extent, were already observed after injecting MABs into a conventional microcatheter [17]. It is conceivable that the multiple smaller MABs themselves do not produce cerebral infarctions or that these infarctions are outside the limit of detection in our radiological and histopathological analyses. The pathophysiology of the scattering and distribution of these multiple MABs causing larger infarctions and the site of the suspected fusion (does it occur at bifurcations or in the terminal vasculature?) is poorly understood and needs to be investigated in future studies.

This experimental study features several special characteristics compared to previous studies. Systematic studies on iatrogenic AE in humans are rare and the ability to analyze the underlying pathophysiological mechanisms is highly limited. Previous experimental studies investigating AE in small animals all used a direct surgical access to the carotid artery [24, 25]. This open surgical intervention bears a risk for the generation of AE to the brain. Only a few large animal studies injected air via conventional catheters via a transfemoral access into the vasculature [26, 27]; however, in these studies, no calibrated air bubbles were used. In contrast to the available previous study, the experimental set-up of our study allowed the injection of a defined number of highly calibrated MABs which were delivered through a conventional microcatheter via a transfemoral access, resembling endovascular interventions in humans. The total volume of injected air was 0.39 µl for 1200 MABs and 0.64 µl for 2000 MABs. Projected to the human brain (assuming a weight of 2 g for the rat brain and 1450 g for the human brain), this volume corresponds to 0.28 mL for 1200 MABs and 0.47 mL for 2000 MABs. This volume of air is comparable to the amount which is released during the deployment of TEVAR stent grafts [28].

Currently, data regarding the pathophysiology of brain damage caused by AE are limited and the mechanisms which lead to the ischemic infarctions is poorly understood. Besides a simple mechanical clogging of the blood vessels, causing ischemia by a disruption of blood supply, the role of inflammatory mechanisms is also discussed [5]. In this study, we observed early signs of ischemic damage but, most probably due to the early stage of the pathophysiological mechanisms 2 h after the injection of the MAB, no substantial inflammatory changes. Studies with longer survival times are needed to explore the pathophysiological mechanisms of AE leading to brain infarctions.

We acknowledge that this study has noteworthy limitations. The number of experiments per study group was small; however, the findings were consistent within the individual study groups. We only used MABs with a size of 85 µm and injected only either 1200 or 2000 MABs. The injection of smaller or larger air bubbles or different numbers of air bubbles could have led to different results. Another limitation is the short follow-up period. Imaging and histopathological analyses after 6–24 h and beyond could have shown a different number and size as well as different histopathological changes of the infarctions. Furthermore, the transfer of an experimental animal model to clinical practice is generally limited.

## Conclusion

Iatrogenic AEs originating at the ascending aorta cause a similar number and embolic pattern of cerebral infarctions compared to those with origin at the carotid artery. Direct injection of MABs into the CCA and a higher number of MABs cause larger brain infarctions. These findings underline the relevance and potential risk of AE occurring during endovascular interventions at the aortic valve and ascending aorta.

### Supplementary Information

Supplemental Table 1: MRI protocol
