# Supplementary material for: Iatrogenic Air Embolisms During Endovascular Interventions: Impact of Origin and Number of Air Bubbles on Cerebral Infarctions
Source: Clin Neuroradiol. 2023 Sep 4;34(1):135–45. doi: 10.1007/s00062-023-01347-2 (PMC10881616; doi:10.1007/s00062-023-01347-2)
Supplement: Supplementary file 1 — Supplemental Table 1: MRI protocol [file 62_2023_1347_MOESM1_ESM.docx]

**Supplemental Table 1:** MRI protocol

| **Sequence** | **TR/TE [ms]** | **Slice thickness [mm]** | **Averages** | **FOV [mm]** | **Matrix Size** | **Flip angle [°]** | **Scan Time  (min:sec)** |
| --- | --- | --- | --- | --- | --- | --- | --- |
| 3D TOF FLASH axial | 12/1.8 | 0.25 | 2 | 30x30x24 | 256x265x96 | 20 | 8:08 |
| 2D T2 Turbo RARE axial | 3667.2/33 | 0.5 | 2 | 35x35 | 256x256 | 90 | 2:56 |
| 3D T2 Turbo RARE axial | 1000/25.89 | 0.1 | 1 | 20x14x20 | 200x140x200 | 90 | 22:00 |
| 2D DWI SE axial | 1332.39/17.58 | 0.5 | 1 | 20x15 | 100x75 | 90 | 10:16 |
| 2D DWI SE cor | 1000/17.57 | 0.5 | 1 | 20x15 | 100x75 | 90 | 7:36 |
| 3D SWI FLASH cor | 50.85/19.30 | 0.08 | 1 | 25x15x12 | 312x188x150 | 12 | 16:55 |
| 2D T1 FLASH pre axial | 298/4 | 0.8 | 3 | 17.5x17.5 | 256x256 | 30 | 2:41 |
| 2D contrast enhanced DSC FID EPI axial | 800/9.9 | 1.2 | 1 | 20x20 | 100x100 | 40 | 2:00 |
| 2D contrast enhanced T1 FLASH post axial | 298/4 | 0.8 | 3 | 17.5x17.5 | 256x256 | 30 | 2:51 |
| 2D T2 Turbo RARE axial | 3667.2/33 | 0.5 | 2 | 35x35 | 256x256 | 90 | 2:56 |
